# Trends in the Incidence and Risk Factors of Pregnancy-Associated Stroke

**DOI:** 10.3389/fneur.2022.833215

**Published:** 2022-04-11

**Authors:** Petra Ijäs

**Affiliations:** Neurology, Helsinki University Hospital and University of Helsinki, Helsinki, Finland

**Keywords:** stroke, cerebrovascular disease, pregnancy, postpartum, prevention

## Abstract

Pregnancy is a female-specific risk factor for stroke. Although pregnancy-associated stroke (PAS) is a rare event, PAS leads to considerable maternal mortality and morbidity. It is estimated that 7.7–15% of all maternal deaths worldwide are caused by stroke and 30–50% of surviving women are left with persistent neurological deficits. During last decade, several studies have reported an increasing incidence of PAS. The objective of this review is to summarize studies on time trends of PAS in relation to trends in the prevalence of stroke risk factors in pregnant women. Seven retrospective national healthcare register-based cohort studies from the US, Canada, UK, Sweden, and Finland were identified. Five studies from the US, Canada, and Finland reported an increasing trend of PAS. Potential biases include more sensitive diagnostics and improved stroke awareness among pregnant women and professionals toward the end of the study period. However, the concurrent increase in the prevalence of several stroke risk factors among pregnant women, particularly advanced age, hypertensive disorders of pregnancy, diabetes, and obesity, indicate that the findings are likely robust and should be considered seriously. To reduce stroke in pregnancy, increased awareness among all medical specialties and pregnant women on the importance of risk-factor management during pregnancy and stroke symptoms is necessary. Important preventive measures include counseling for smoking cessation and substance abuse, treatment of hypertensive disorders of pregnancy, use of aspirin in women at high risk for developing preeclampsia, and antithrombotic medication and pregnancy surveillance for women with high-risk conditions. Epidemiological data from countries with a high risk-factor burden are largely missing. National and international registries and prospective studies are needed to increase knowledge on the mechanisms, risk factors, management, and future implications for the health of women who experience this rare but devastating complication of pregnancy.

## Introduction

**Figure 1:**
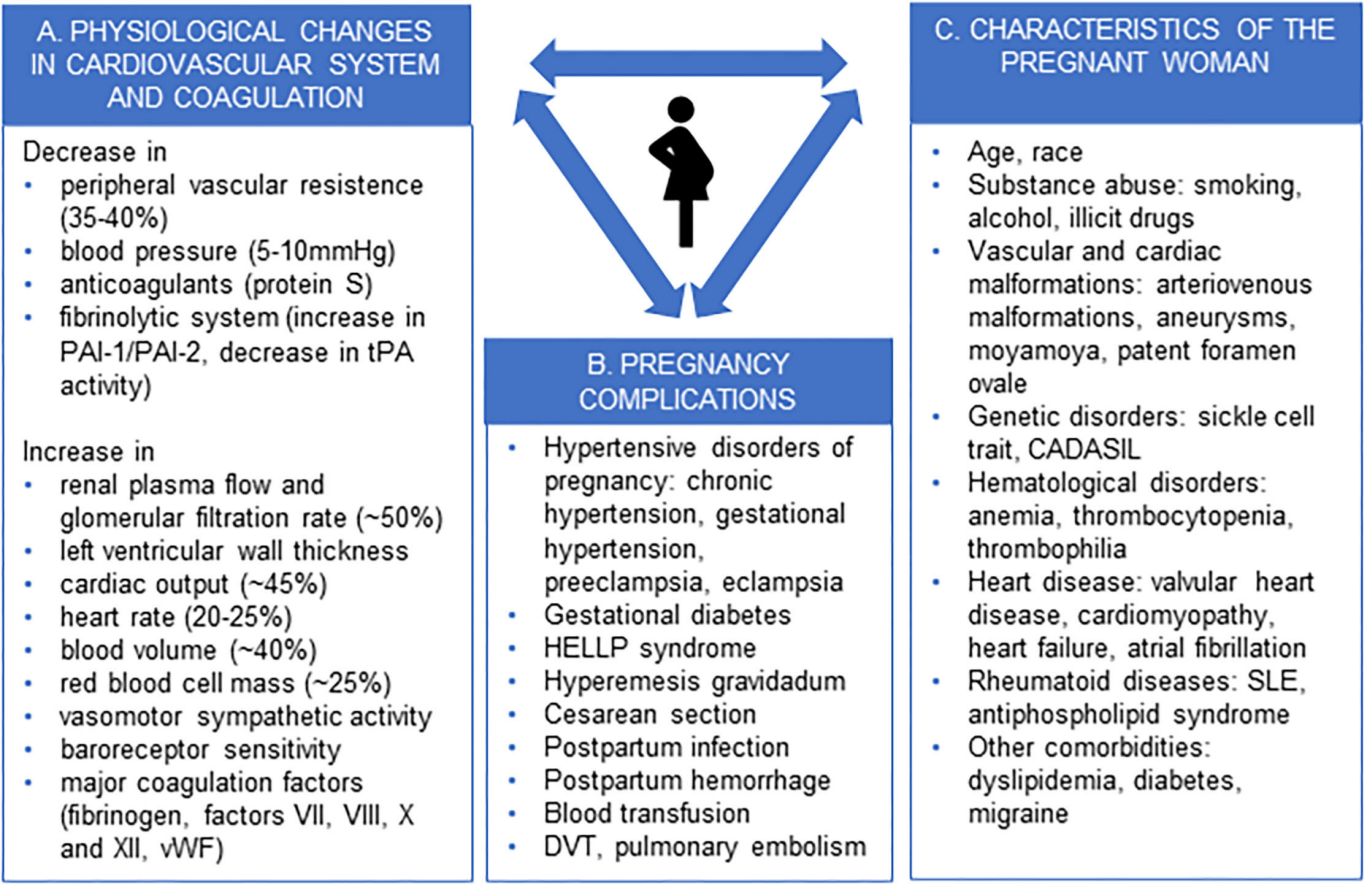
Physiological changes in pregnancy **(A)**, pregnancy complications **(B)**, and characteristics of the pregnant woman **(C)** predisposing to stroke. PAI-1, plasminogen activator inhibitor-1; PAI-2, plasminogen activator inhibitor-2, tPA, tissue plasminogen activator; vWF, von Willebrand factor; HELLP, haemolysis, elevated liver enzymes and low platelets, DVT, deep venous thrombosis, CADASIL, cerebral autosomal dominant arteriopathy with subcortical infarcts and leukoencephalopathy, SLE, systemic lupus erythematosus.

Pregnancy, along with postpartum period (puerperium), is a female-specific risk factor for stroke (1). Pregnancy-associated stroke (PAS) accounts for 18% of strokes in women aged <35 years (2). During pregnancy, the female body undergoes significant physiological changes to adapt to the growth of the fetus and to prepare for delivery (Figure 1A) (3–5). Many of these changes may render the woman more vulnerable to thromboembolism and cardiovascular events to such an extent that pregnancy has been called Nature's stress test (3, 5). Pregnancy and its complications may reveal pre-existing maternal characteristics and comorbidities that predispose to cardiovascular diseases and stroke, such as heart disease, genetic and coagulation disorders, and malformations of cerebral vasculature (Figures 1B,C) (3, 5, 6).

Although a rare event, PAS accounts for considerable maternal mortality and morbidity. Approximately 7.7–15% of all maternal deaths worldwide are caused by stroke, with the highest mortality related to intracerebral hemorrhage (ICH) (7, 8). The estimated case fatality rate is 13.8% for ICH and 3.9% for ischemic stroke (9). Furthermore, stroke is a major cause of disability, as 30–50% of the mothers who experience stroke have persistent neurological deficits, which subsequently affects their ability to care for themselves and the newborn, to participate in family life, and to return to work (10).

Published estimates on the incidence of PAS are highly variable and range from 3.8 to 98.4 per 100 000 hospitalizations. This can be explained by differences between studies regarding source data (single institution, pregnancy or stroke registry, insurance or healthcare registry, questionnaire-based), inclusion criteria (stroke types, antenatal/postpartum), and geographical and population factors (healthcare system and income of the country, population genetics) (6, 11). In a recent meta-analysis including 11 studies from seven countries, the incidence of pregnancy-related stroke was estimated as 30.0 per 100 000 pregnancies (95% confidence interval [CI] 18.8–47.9), which is approximately 2–3 times greater than the rate in non-pregnant young adults (11). The rates were approximately equal between ischemic stroke (12.2, 95% CI 6.7–22.2), cerebral venous thrombosis (CVT) (9.1, 95% CI 4.3–18.9), and haemorrhagic stroke (12.2, 95% CI 6.4–23.2). The crude stroke rate was 18.3 (95% CI 11.9–28.2) for antenatal/perinatal stroke and 14.7 (95% CI 8.3–26.1) for postpartum stroke.

During the last decade, reports on the increasing incidence of stroke during pregnancy and puerperium have been published from several countries (12–17). The objective of this non-systematic review is to summarize the results from these recent studies on the incidence trends of PAS and to discuss the findings in relation to trends on the prevalence of stroke risk factors in pregnant women. Potential areas for future research are discussed.

## Methods

This was a non-systematic or narrative review. For the review on incidence trends of PAS, an electronic search included PubMed, Medline, and Google Scholar and used the search terms ‘stroke’, and ‘pregnancy’ or ‘postpartum’ or ‘puerperium’ and ‘incidence’. Titles and (if required) abstracts were screened for relevant articles. Original articles reporting incidence rate trends in time for stroke during pregnancy or the postpartum period up to 12 weeks after delivery were included in the review. The exclusion criteria were articles for which full text was not available, articles not in English, or articles that reported incidence data only for a restricted subpopulation of pregnant women (e.g., women with hypertensive disorders of pregnancy or women with subarachnoid hemorrhage). From the articles retrieved in the first search round, additional references were identified by a manual search among the cited references. For the review on trends in the prevalence of stroke risk factors, an additional electronic search was performed using the search terms ‘stroke’ and ‘pregnancy’ or ‘postpartum’ or ‘puerperium’ and ‘risk factors’. Additional references were identified by a manual search among the cited references from both searches. Searches were performed in December 2021.

## Incidence Trends of Pregnancy-Associated Stroke

The literature search identified eight studies. Three were from the Nationwide Inpatient Sample in the US (12–14), two were national healthcare register-based studies from Canada (15), and one each were from the UK (18), Sweden (19), and Finland (16). One study on temporal trends of severe maternal morbidity in Canada (20) was excluded as it analyzed the same dataset and time period as the study specifically focused on PAS (15). The remaining seven studies are summarized in Table 1.

**Table 1:** Studies reporting long-term incidence trends of pregnancy-associated stroke.

**Country**	**Study years**	**Data source; N**	**Outcome**	Change during the study period^*^
US (12)	1994–95 vs. 2006–07	Nationwide Inpatient Sample; not specified	Hospitalizations with antenatal or postpartum stroke, incl. TIA	Antenatal: 15 to 22 per 100 000 deliveries (*p* < 0.001); postpartum: 12 to 22 per 1000,000 deliveries (*p* < 0.001)
US (13)	1994–95 vs. 2010–11	Nationwide Inpatient Sample; 81,983,216 pregnancy hospitalizations	Pregnancy-related stroke hospitalizations with or without HDP, incl. TIA	Stroke with HDP: 8 to 16 per 100 000 hospitalizations (*p* < 0.001); stroke without HDP: 22 to 32 per 100,000 hospitalizations (*p* < 0.001)
US (14)	2007 vs. 2015	Nationwide Inpatient Sample; 37,360,772 pregnancy hospitalizations	Pregnancy-related acute stroke hospitalizations, incl. TIA	TIA included: 42.8 to 42.2 per 100 000 hospitalization (*p* = 0.10); TIA excluded: 29.8 to 33.0 per 100,000 hospitalizations (*p* < 0.0001)
Canada (15)	2003–04 vs. 2015–16	Canadian Institute of Health Information; 3,907,262 deliveries	Pregnancy-related stroke hospitalizations, incl. TIA and other cerebrovascular diseases	10.8 to 16.6 per 100,000 deliveries (*p* = 0.002)
Sweden (19)	1992–96 vs. 2007–12	National healthcare registers (Medical Birth Register, National Patient Registry); 1,124,541 women	Incidence rates for first incident stroke per 100,000 person-years, IRR for pregnancy periods and non-pregnant time, excl. TIA	Peripartum or early postpartum stroke: 106.5 to 93.5 per 100,000 person-years^†^
UK (18)	1997–2002 vs. 2009–14	National healthcare registers (Clinical Practice Research Datalink, Hospital Episode Statistics); 2,046,048 women	Incidence rates for first incident stroke per 100 000 person-years, IRR for pregnancy periods and nonpregnant, excl. TIA	49.8 to 59.7 per 100,000 person-years^†^
Finland (16)	1987–91 vs. 2012–16	National healthcare registers (Medical Birth Register, Hospital Discharge Register, Register of Causes of Death), cases chart-verified; 1,773,728 deliveries	Incident stroke per 100 000 deliveries, excl. TIA	11.1 to 25.2 per 100 000 deliveries (*p* < 0.0001)

*HPD, hypertensive disorders of pregnancy; IRR, incidence rate ratio*.

* *p-value from time-trend analysis reported in the study*.

† *Time-trend analysis was not performed in the study*.

All studies from the US utilized data on pregnancy-related stroke hospitalizations in the Nationwide Inpatient Sample database, the largest all-payer, publicly available database of inpatient hospitalizations in the US. Their results cannot be directly compared due to different study outcomes and time periods between 1994 and 2015 (12–14). The earliest study that compared incidence rates of hospitalizations with antenatal or postpartum stroke between 1994–95 and 2006–07 reported increases both in antenatal strokes (from 15 to 22 per 100 000 deliveries, *p* < 0.001) and postpartum strokes (from 12 to 22 per 100,000 deliveries, *p* < 0.001) (12). A later study investigated the effect of hypertensive disorders of pregnancy (HDP) on the risk of stroke during pregnancy and noted that the incidence of stroke increased significantly both in women with and without HDP (13). In the most recent study, the incidence of acute stroke and TIA remained relatively unchanged between 2007 and 2015 (14). However, in the secondary analysis, in which TIA and pregnancy-specific codes were excluded, the incidence of acute stroke increased from 29.8 to 33.0 per 100 000 pregnancy-related hospitalizations (*p*_trends_ < 0.0001).

In the data from the Canadian Institute of Health Information, the incidence of stroke, TIA, and cerebrovascular disease rose from 10.8 per 100 000 in 2003–04 to 16.6 per 100 000 deliveries in 2015–16 (*p* = 0.002) (15). Most cases were haemorrhagic strokes (58.6%) and occurred in the postpartum period (51.5%). This study included a wide spectrum of diagnostic codes related to cerebrovascular disease and it was not possible to verify whether the identified strokes were first-time events or complications of an earlier event. Nevertheless, the incidence was lower than in the earlier studies from the US. A recent Finnish population-based study that covered a 30-year time period from 1987 to 2016 revealed an increasing incidence of PAS from 11.1 to 25.2 per 100 000 deliveries from 1987–91 to 2012–16 (*p* < 0.0001) (16). Among stroke subtypes, the rising trend was significant for ischemic stroke and CVT but not for subarachnoid hemorrhage (SAH) or ICH. The main strength of this study was that the stroke cases were verified from medical records, resulting in the exclusion of 70% of register-identified cases. The main reasons for exclusion were history of stroke not associated with pregnancy (36.6%), neurologic symptoms and suspicion of stroke during pregnancy or postpartum period leading to an alternative diagnosis after evaluation (stroke mimics, 17.2%), and anomalies of cerebral vasculature without an acute cerebrovascular event (9.5%).

Two other studies, one from Sweden (19) and another from the UK (18), examined incidence rates during an approximate 20-year time period but did not specifically report on trends in time. The incidence rates reported from the UK for the 5-year time periods 1997–2002 and 2009–2014 increased slightly (18). In the Swedish study, which reported only peripartum or early postpartum stroke, a decreasing incidence was revealed (19).

### Summary

Seven studies on incidence trends of PAS over time were identified by the literature search. All the studies specifically addressing incidence trends over time, reported an increasing incidence of PAS. Studies were retrospective cohort studies from national healthcare registers, where stroke cases were identified by stroke ICD codes. One study verified diagnoses from medical records. The Finnish and Swedish registers are nationwide. The Canadian register included hospitalizations from all Canadian provinces except Quebec. The US Nationwide Inpatient Sample is the largest all-payer, publicly available database of inpatient hospitalizations in the US and includes all discharge data from 1,050 hospitals in 44 states, approximating a 20% stratified sample of US community hospitals. Thus, the register-based studies have good coverage and generalizability over the studied populations. The incidence rates are difficult to compare due to different diagnostic inclusion criteria and incidence rate definitions. However, the incidence rates in Canada and Finland appear lower than those in the US. Studies on incidence trends were only found from a few high-income countries, and data from middle- and low-income countries are lacking.

## Trends in the Prevalence of Risk Factors of Pregnancy-Associated Stroke

Stroke during pregnancy and postpartum often arises from an adverse interaction between normal physiological changes related to pregnancy, complications of pregnancy or delivery, and baseline characteristics of the pregnant woman (Figure 1) (6, 14, 16, 21, 22). Lifestyle factors and diseases of the pregnant woman that increase stroke risk include substance abuse (smoking, alcohol, illicit drugs), obesity, diabetes, dyslipidaemia, chronic hypertension, heart disease, migraine, antiphospholipid syndrome and systemic lupus erythematous, coagulopathy, certain genetic traits (sickle cell disease, CADASIL), and cerebrovascular malformations (aneurysms, arteriovenous malformations, moyamoya disease) (6, 14, 16, 21, 22). Among demographic factors, advanced age and African-American race are associated with an increased risk of PAS (14–16, 21). Complications of pregnancy and delivery, such as hyperemesis gravidarum, post-partum hemorrhage and infection, and Cesarean section, may precipitate stroke by exaggerating a prothrombotic state and fluid-electrolyte-acid-base disturbances (21, 22).

The prevalence of several known risk factors for PAS is increasing. Age is the most important non-modifiable risk factor for stroke and also increases the risk of stroke in pregnancy. In the Finnish population-based study, the incidence was three times higher in women >40 years than in those between 20 and 24 years (16). In many middle- and high-income countries, the mean maternal age at first birth has increased by several years. In the US, the percentage of first births for women 40–44 years increased by 70% between 1991 and 2001 (23). Advanced age increases the risk of stroke by multiple mechanisms. Women with advanced age typically have more classical vascular risk factors and may be less able to adapt to pronounced cardiovascular changes related to pregnancy, which renders them more vulnerable to pregnancy complications, particularly HDP (24). Less evident associations have also been described; a systematic review and meta-analysis of long-term cardiovascular effects of fertility therapy revealed a trend of a higher incidence of stroke (HR 1.27; 95% CI 0.96–1.68). It is not known if this applies to PAS.

Several modifiable stroke risk factors have also become more common among pregnant women. Obesity among pregnant women is a global problem, particularly in upper middle and lower middle income countries, where sharp increases in the proportions of overweight and obese pregnant women have been observed. In 2014, one fifth of women in India and a third of women in the US were obese (25). At the same time, an over 2-fold increase in the prevalence of pre-existing diabetes in pregnant women, particularly among younger women, was noted between 1999 and 2005 in the US (26). Overweight women have significantly higher blood pressure at any point during the pregnancy and postpartum than women with lower body mass and are more prone to develop HDP (27). These trends were also observed in the reviewed incidence trend studies. In the earliest study by Kuklina and colleagues, the increases in the prevalence of HDP and heart disease were considered to almost exclusively explain the increase in postpartum hospitalizations (12). In the later study on the Nationwide Inpatient Sample database, there was an increase in the prevalence of obesity, smoking, hyperlipidaemia, migraine, atrial septal defects, prior stroke, and gestational hypertension among women with pregnancy-associated acute stroke or TIA (14). The interaction between many risk factors, such as obesity, diabetes, and HDP, may lead to clustering of several minor stroke risk factors, while pregnancy acts as a trigger for stroke.

Probably the most significant risk factor for PAS worldwide is HDP, which includes pre-existing hypertension (chronic hypertension), hypertension developing after week 20 in pregnancy (gestational hypertension), and preeclampsia/eclampsia (28). The reported prevalence of preeclampsia/eclampsia among women with PAS varies between 73% in India (29), 47% in France (10), 31% in the US (13), and 22% in Taiwan (9). HDP is associated with all stroke subtypes (9, 10, 16, 30). Women with HDP are approximately five-times more likely to have a stroke than those without; the presence of traditional stroke risk factors further increased this risk (13). Women hospitalized with HDP and stroke also had higher rates of complications than women without HDP, including the need for mechanical ventilation, seizure, pneumonia, prolonged hospital stay, and death during hospitalization (13). In data from the Global Burden of Disease 2019 Study, covering populations from 204 countries and territories, the total number of incident cases of HDP increased by 10.92% from 1990 to 2019 but the age-standardized incidence rate decreased (31). The authors suggested that this is related to population growth, advanced maternal age, and multiple pregnancies. The highest incidence rates of HDP were found in South Asia, western sub-Saharan Africa, and eastern sub-Saharan Africa.

The prevalence of several other potential risk factors for PAS is also increasing. Heart disease is an important risk factor for stroke (14, 32). Although the number of persons with heart diseases is not increasing due to better management, they more often become pregnant and thus encounter the risks associated with pregnancy. The same may apply to other patients with severe pre-existing conditions. Cesarean section is more commonly used and may promote thrombotic events (22).

### Summary

The prevalence of several well-known risk factors for PAS has increased worldwide. The age of first-time mothers has increased, when they are more likely to have pre-existing comorbid conditions and are also more susceptible to pregnancy complications, notably HDP. The increasing incidence worldwide of obesity and diabetes also increases the risk of HDP and thromboembolism. Advancements in the management of certain medical conditions, such as heart and autoimmune diseases, increase the number of women with high-risk conditions who wish to become pregnant. Collectively, these factors may contribute to the increasing trend of PAS.

## Discussion

The incidence of PAS and its major risk factors has shown increasing trends during the last few decades. The incidence of stroke during pregnancy and puerperium is increasing in several high-income countries (12–16). Epidemiological data on pregnancy-associated stroke from many countries with high risk factor burden (25, 31) is largely missing. Older age accompanied with major stroke risk factors, such as hypertensive disorders of pregnancy, obesity, and diabetes, is becoming more prevalent among pregnant women.

There may be some alternative explanations for the incidence findings. It cannot be excluded that the identification of stroke cases has become more accurate over time. The general awareness of stroke and its symptoms has improved since the 1990s and thus there may be a lower threshold to seek medical attention for neurological symptoms (33). Furthermore, the development of efficient acute treatments for stroke has likely prompted clinicians to refer their patients with suspected stroke to specialists. Accordingly, patients with minor stroke may be more readily diagnosed. More sensitive imaging-based diagnostics by magnetic resonance imaging (MRI) are increasingly available, even in smaller hospitals in rural areas. This is particularly apparent in the case of minor stroke and local CVT, where diagnosis would not be possible without MRI (34). However, in the case of ischemic stroke, MRI can more readily diagnose stroke mimics and functional neurological disorders in addition to minor strokes. This was reflected in the Finnish population-based study, where 17% of the register-identified cases were excluded as stroke mimics after chart review (16). Evidence for a real increase in the incidence of PAS is the concomitant prominent increasing trends in stroke risk factors in pregnant women.

The improved general management of stroke and declining trend of stroke in older age groups suggests that the increasing prevalence of PAS can be reversed. For example, the Tromsø study showed that changes in cardiovascular risk factors explained 57% of the decrease in ischemic stroke incidence in the general population from 1995 to 2012. Reduction in systolic blood pressure and prevalence of smoking accounted for 26 and 17% of the observed decline, respectively (35). Since younger women are generally considered at low risk of cardiovascular diseases, the recognition of pregnancy as a stroke risk factor requires education of women and healthcare professionals working with pregnant women to achieve similarly efficient screening and management of blood pressure and other stroke risk factors in pregnant women.

Very little research data and no randomized controlled trials exist on preventative treatment or acute management of stroke in pregnancy. Despite the rising incidence, stroke in pregnancy remains rare and it is highly unlikely that randomized controlled trials will be conducted. In recent years, guidelines and statements based on case series and expert opinion have been published in the US (36) and Canada (37, 38). Furthermore, extensive international and national guidelines exist on the management of HDP (28), diabetes (39), and cardiovascular diseases (32, 40) during pregnancy. As a general guideline, counseling for women with pre-existing cardiovascular disease should begin before pregnancy. Such women should be managed by multidisciplinary teams, high-risk patients should be treated in specialized centers, and diagnostic procedures and interventions should be performed in centers of expertise. Low-threshold maternal services and preventive pregnancy surveillance systems are important for screening and identifying women at high risk of PAS. Important measures of prevention include counseling for smoking cessation, screening and treatment of HDP, use of aspirin in women at high risk for developing preeclampsia, and antithrombotic medication and pregnancy surveillance for women with high-risk conditions (such as thrombophilias, heart diseases, previous stroke, or cerebrovascular malformation). Follow up after delivery should continue for at least 6 weeks or women should haver low-threshold maternal services to contact if neurological symptoms develop after delivery.

Several open questions and knowledge gaps remain. Very little is known on the exact pathogenic mechanisms responsible fo acute stroke during pregnancy and puerperium; such knowledge is needed to improve treatment. Preeclampsia is an established risk factor for long-term cardiovascular disease and stroke, and guidelines recommend following women with preeclampsia or gestational hypertension for cardiovascular risk factors after delivery (36, 38). Whether the increased risk of future stroke and cardiovascular disease applies to women with PAS and TIA is not known but is plausible. This suggests that more rigorous follow-up and management of women with PAS is necessary. National and international registries and prospective studies, such as the SiPP study (41), are urgently needed to improve knowledge on this rare but devastating complication of pregnancy.

## Author Contributions

The author confirms being the sole contributor of this work and has approved it for publication.

## Conflict of Interest

The author declares that the research was conducted in the absence of any commercial or financial relationships that could be construed as a potential conflict of interest.

## Publisher's Note

All claims expressed in this article are solely those of the authors and do not necessarily represent those of their affiliated organizations, or those of the publisher, the editors and the reviewers. Any product that may be evaluated in this article, or claim that may be made by its manufacturer, is not guaranteed or endorsed by the publisher.
